# High-operating-temperature mid-infrared photodetectors via quantum dot gradient homojunction

**DOI:** 10.1038/s41377-022-01014-0

**Published:** 2023-01-01

**Authors:** Xiaomeng Xue, Menglu Chen, Yuning Luo, Tianling Qin, Xin Tang, Qun Hao

**Affiliations:** 1 School of Optics and Photonics, Beijing Institute of Technology, No. 5 Zhongguancun South Street, Beijing, China; 2 Beijing Key Laboratory for Precision Optoelectronic Measurement Instrument and Technology, Beijing, China; 3 Yangtze Delta Region Academy of Beijing Institute of Technology, Beijing, China

**Keywords:** Photonic devices, Imaging and sensing, Nanophotonics and plasmonics

## Abstract

Due to thermal carriers generated by a narrow mid-infrared energy gap, cooling is always necessary to achieve ideal photodetection. In quantum dot (QD), the electron thermal generation should be reduced with quantum confinement in all three dimensions. As a result, there would be a great potential to realize high-operating-temperature (HOT) QD mid-IR photodetectors, though not yet achieved. Taking the advantages of colloidal nanocrystals’ solution processability and precise doping control by surface dipoles, this work demonstrates a HOT mid-infrared photodetector with a QD gradient homojunction. The detector achieves background-limited performance with D^*^ = 2.7 × 10^11^ Jones on 4.2 μm at 80 K, above 10^11^ Jones until 200 K, above 10^10^ Jones until 280 K, and 7.6 × 10^9^ Jones on 3.5 μm at 300 K. The external quantum efficiency also achieves more than 77% with responsivity 2.7 A/W at zero bias. The applications such as spectrometers, chemical sensors, and thermal cameras, are also approved, which motivate interest in low-cost, solution-processed and high-performance mid-infrared photodetection beyond epitaxial growth bulk photodetectors.

## Abstract (web summary)

Colloidal quantum dot gradient homojunction would effectively improve the detectivity of mid-infrared photodetector at high-operating temperatures, motivating interest in low-cost, solution-processed and high-performance mid-infrared photodetection.

## Graphical abstract

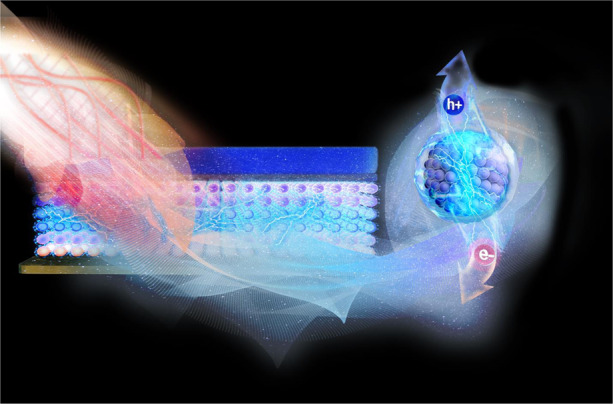

## Introduction

Mid-infrared (mid-IR) is of interest for photodetection because of not only matches the atmosphere window but also gives additional thermal information compared with visible or near-infrared. The main commercial photodetectors in this wavelength are based on epitaxial materials such as HgCdTe bulk and GaAs/AlGaAs quantum well^1^. However, according to Boltzmann’s equation, high thermal carrier density would be easily generated by the narrow energy gap, where cooling is always necessary to achieve ideal device performance. Theoretically, the quantum-mechanical nature of quantum dot (QD) implies higher operating temperature on infrared photodetectors, since thermal carrier generation would be significantly reduced compared to a quantum well due to the energy quantization in all three dimensions^2^. Epitaxial QD has been investigated first. There are reports of the enhancement of epitaxial QD infrared photodetector performance with heterostructure designs^3^, multiple periods of QD active layers^4^, resonant cavity enhancement^5^, and photonic crystal cavity^6^ enhancement. The reported detectivity of 10^11^ cmHz^1/2^/W could be achieved at 3.9 μm at 100 K, however, would drop quickly with increasing temperature^4^. Besides, the complex fabrication of epitaxy materials^7^ challenges the wide range of investigations and applications. Colloidal quantum dot (CQD), taking the advantages of cheap chemical synthesis and solution processability^8,9^, could provide an ideal platform for QD photodetector investigations^10^. In the last 10 years, CQDs have been successfully applied for mid-IR photodetection^11^, such as photoconductor^12,13^, phototransistor^14^, heterojunction^15,16^ photovoltaic devices^17–19^, optical structures enhanced devices^20^, and dual band photovoltaic devices with multijunction^18,21^. The background-limited performance (BLIP) has been achieved for mid-IR CQD photodetectors at cryogenic temperature^18,20^. Still, the predicted high-operation-temperature (HOT) has not yet been achieved. What’s more, the sparse CQD packing^22^ causes the low absorption of input photons. As a result, the low external quantum efficiency (EQE)^17,18^ is much lower compared to the epitaxial growth HgCdTe detector^23^ for mid-IR. In our opinion, using the surface ligand modification^24^ to control over CQD packing density, as well as transport properties like carrier type, density, and mobility, might be the key. For example, for near-IR wavelength, improving carrier mobility helps achieving ultrahigh photoconductivity^25^ while the controllable carrier types^26,27^ and density would contribute to advanced architectures like homojunction^28,29^. Here, HgTe CQDs are used as mid-IR optoelectronic material. With a solution phase ligand exchange^30^, the carrier mobility^31^ could be above 1 cm^2^ V^−1^ s^−1^. More importantly, precisely controllable n-type, intrinsic and p-type doping mid-IR CQD ink could be obtained with additional surface dipoles. We also demonstrate PIN gradient homojunction based on mid-IR HgTe CQD with the specific detectivity 2.7 × 10^11^ Jones on 4.2 μm at 80 K, above 10^11^ Jones until 200 K, above 10^10^ Jones until 280 K, and 7.6 × 10^9^ Jones on 3.5 μm at 300 K. Besides, the EQE reaches above 77% with the responsivity 2.7 A/W at 80 K. The applications like spectrometers and chemical sensors, are also approved, promoting low-cost but high-performance mid-IR photodetectors.

## Results

**Fig. 1:**
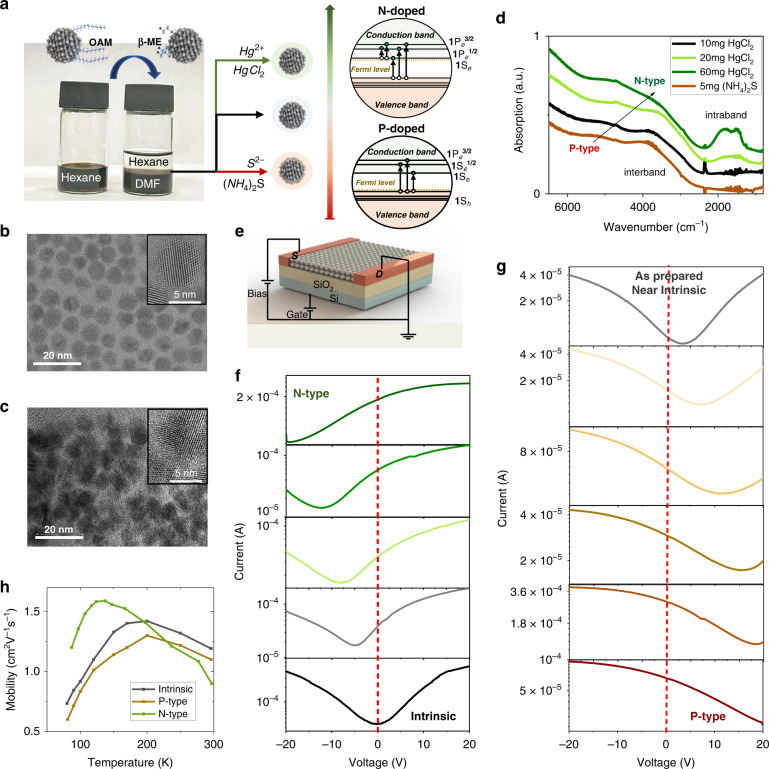
HgTe CQD. **a** Phase transfer and Doping control. **b** TEM of OAM capped HgTe CQD. **c** TEM of HgTe CQD after phase transfer. **d** Absorption spectra of CQD with different doping. **e** Schematic diagram of FET device. **f**, **g** FET transfer curves of p-type, n-type and intrinsic on CQD solids at room temperature. **h** Mobility as a function of temperature

Recent hybrid ligand exchange for HgTe CQD has realized high mobility >1 cm^2^ V^−1^ s^−1^ and tunable n-type doping by additional HgCl_2_ salt during the ligand exchange process^30^. However, the roles of these hybrid ligands remain unclear and the method of realizing tunable p-type doping in HgTe CQD solid is still lacked^13^. Here, with a modified ligand exchange method, we achieve high mobility CQD solids larger than 1 cm^2^ V^−1^ s^−1^, with precisely controllable n-type or p-type doping by adding additional HgCl_2_ or (NH_4_)_2_S salt. The modified ligand exchange method includes liquid phase ligand exchange, doping modification, and solid-phase ligand exchange with Fermi level fixing. As shown in Fig. 1a, β- mercaptoethanol (β-ME) is used to replace the original long-chain oleyamine (OAM) ligands on CQDs, since Hg-S bond is much stronger than Hg-OAM. At the same time, hydroxyl in β-ME would promote CQDs transferred from hexane to *N*,*N*-Dimethylformamide (DMF) and stabilized. The ligand exchange in the liquid phase, effectively reduces CQD spacing, as shown in TEM (Fig. 1b, c). Ammonium surfactants, dimethyldioctadecylammonium bromide (DDAB), forming unilamellar vesicles in polar solution, could be used to accelerate this liquid phase transfer. We note the CQD dissolved in DMF is called CQD ink. CQD solids are prepared by spin-coating and followed by solid-phase ligand exchange with 1,2-Ethanedithiol (EDT) and HCl, which is necessary for solid stability (Fig. S1, SI).

N-type, intrinsic, and p-type CQD ink could be prepared by additional surface dipoles introduced by HgCl_2_ or (NH_4_)_2_S salt^32,33^. The tunable doping is first approved by absorption spectra (Fig. 1d). The extra HgCl_2_ salt produces n-type CQD, which would induce intraband absorption at ~1830 and 1500 cm^−1^. The splitting in the intraband transition comes from the spin–orbital coupling^34,35^, also demonstrating the induced transition from quantum state-resolved n-type doping in the conduction band rather than the plasmonic resonance. The extra (NH_4_)_2_S would introduce p-type doping, though not obvious in absorption spectra, which could be figured out by the field effect transistor (FET). Figure 1f, g show the room temperature transfer curves of different CQD solids. The as-prepared solid is near intrinsic, while the very intrinsic solid could be obtained by adding 10 mg additional HgCl_2_ during the phase transfer process. For the n-type solid, more HgCl_2_ could be added, while (NH_4_)_2_S would be added for the p-type solid (Methods). Carrier density is estimated by FET measurement (Methods), indicating a tunable doping range from 1.6 h/dot to 1.7 e/dot. The quantum state filling is not resolved here since the measurement is done at room temperature. State-resolved filling in FET is observed at cryogenic temperature (Fig. S2, SI). The FET mobility ($\mu ^{{\mathrm{FET}}}$), extracted in the linear regime, was calculated by fitting the transfer curve (Methods). As shown in Fig. 1g, all CQD solids with different carrier density and type show similar high mobility ~1 cm^2^ V^−1^ s^−1^ at the temperature range between liquid nitrogen and room temperature. This feature would benefit the junction design by avoiding carrier mobility mismatch between differently doped layers.

**Fig. 2:**
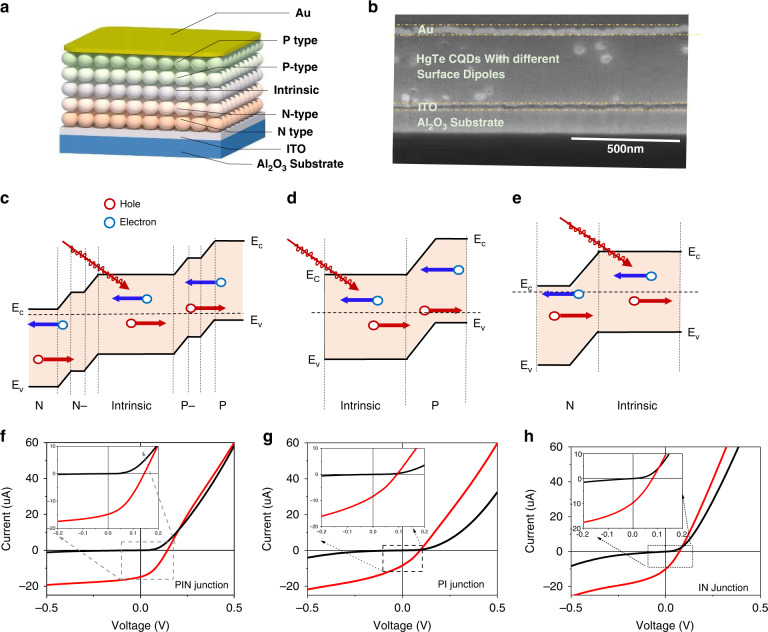
CQD photodiodes. **a** Schematic diagram of high mobility CQD homojunction photodiode. **b** SEM of high mobility CQD homojunction photodiode cross-section. **c**–**e** Energy diagram of high mobility PIN gradient, PI, and IN homojunction, respectively. **f**–**h** I-V curve characterization on high mobility PIN gradient, PI, and IN homojunction devices with the insert graph zoom in the near zero bias part at 80 K, respectively

The schematic diagram and SEM cross-section of the homojunction PV device are shown in Fig. 2a, b. The device is fabricated on an Al_2_O_3_ substrate. About 50 nm indium tin oxide (ITO) layer with a working function around 4.5 to 4.7 eV^36^ is served as the electron contact, which could minimize electron transport since it is compatible with the HgTe CQD conduction band around 4.5 eV. About 50 nm gold would be served as the top contact. For PIN gradient homojunction, ~60 nm thickness n-type (doping at 0.8 e/dot), ~40 nm thickness n-type (doping ~0.1 e/dot), ~300 nm thickness intrinsic, ~40 nm p-type (doping ~0.1 h/dot) and ~60 nm p-type (doping ~0.8 h/dot) CQD solids are stacked before ~50 nm gold contact deposited. As a reference, PI junction and IN junction are prepared. For PI junction, 340–350 nm and 120 nm in thickness intrinsic and p-type HgTe CQD solid are deposited in series. For IN junction, 120 nm and 340–350 nm n-type and intrinsic HgTe CQD solid are deposited in series. The energy diagram of high mobility PIN gradient, PI and IN homojunction PV are shown in Fig. 2c–e. Although the different self-doped CQD solid layers are unobvious in the SEM cross-section, the devices show different IV curve characterizations at 80 K, as shown in Fig. 2f–h. The open circuit voltages are 143, 96, and 73 mV for PIN gradient, PI, and IN homojunctions, respectively. The gradient doping layers provide a much better band alignment, resulting in a much stronger junction and more than 50% larger open circuit voltage. This would benefit the extraction of carriers and suppress dark current, as shown in the inserted graph in Fig. 2f–h.

## Discussion

### Photodiode characterization

**Fig. 3:**
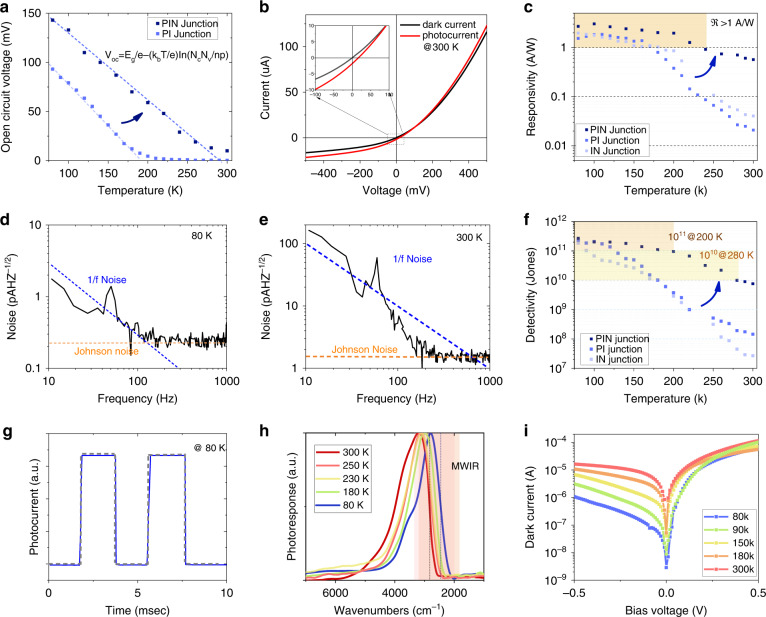
PV device characterization. **a** Open circuit voltage as a function of temperature on PIN gradient and PI homojunctions. **b** IV characterization of PIN gradient homojunction at 300 K. **c** Responsivity as a function of the temperature of PIN gradient, PI, and IN homojunction devices. **d**, **e** Noise spectra of the detector at 80 and 300 K of PIN gradient homojunction devices. **f** D^*^ as a function of the temperature of PIN gradient, PI, and IN homojunction devices. **g** Response speed. Photoresponse was measured with the incident light modulated at 500 Hz. **h** Photoresponse spectra of high mobility homojunction PV at different temperatures. **i** Dark currents of the PIN junction device vary with temperature

Open circuit voltage could be described as

1 $$V_{oc} = \frac{{E_g}}{e} - \frac{{k_bT}}{e}{{{\mathrm{ln}}}}\left( {\frac{{N_cN_v}}{{np}}} \right)$$

where *E*_*g*_ is the energy gap, *k*_*b*_ Boltzmann constant, T temperature, e elemental electron charge, *N*_*c*_ (*N*_*v*_) conduction (valence) band density ~$2\left| e \right|/dot$, *n* (*p*) electron(hole) density. As shown in Fig. 3a, the slope of $V_{oc}$ as a function of temperature in the PIN gradient homojunction (dark blue dot) is smaller compared to the PI homojunction (pale blue dot). The experimental results give $\frac{{N_cN_v}}{{np}}$ = 550 for PIN gradient homojunction and $\frac{{N_cN_v}}{{np}}$ = 2000 for PI homojunction. Take the PI junction as a reference, if we assume electron carrier density is equal to and hole carrier density, n = p ~0.045 $\left| e \right|/dot$. However, if we consider that the FET measurement giving p-type layer doping density ~0.8 holes/dot, n should be ~0.0025 e/dot. Then, one could estimate the depletion length $d = \sqrt {\frac{{2\varepsilon _{eff}\varepsilon _0V_{oc}}}{e}(\frac{1}{n} + \frac{1}{p})}$, which is about 165 nm. Here, $\varepsilon _{eff} = 8.4$ is the effective dielectric constant from the measured optical index, which will be discussed later. Obviously, the depletion length is much thinner compared to the device thickness, which is about 470 nm as described. Those extracted values on depletion length and doping density inspired us on the design of PIN gradient homojunction, where strong n-type, weak n-type, intrinsic, weak p-type, and strong p-type CQD solids are stacked in series. The doping of a strong n or p-type layer is ~0.8|e|/dot, which provides large enough band shift to build a strong junction. The thickness of the strong doped layer is about 60 nm, much larger than the depletion length inside the doped layer, which is about 10 nm. However, the thicker, strong doped layer is necessary to prevent oxidation and impurities since all the fabrication steps are done in the ambient environment. The doping of weak n or p-type layer is ~0.1|e|/dot with a thickness is 40 nm. As discussed above, *n* = *p* ~0.045 $\left| e \right|/dot$ assuming electron and hole carrier density is equal, so slightly higher doping level is chosen for this weak doped layer, while the thickness is chosen considering the depletion length inside the weak doped layer. The thickness of the very intrinsic CQD layer is about 300 nm. The strategy for the gradient homojunction design is to obtain large internal electric field, which clearly works. On the one hand, the open circuit voltage $V_{oc}$ is much improved in the PIN gradient homojunction, which is 143 mV at 80 K, more than 50% larger than the PI or IN homojunction as shown in Fig. 2g, h. For mid-IR QD detector, $V_{oc}$ usually drop quickly with temperature increasing, which is close to zero above 200 K. Here with precisely doped layers, the diode would show decent performance where $V_{oc}^{200K}$ is above 60 mV for PIN gradient homojunction, much larger than PI junction’s $V_{oc}$ which is 3 mV at 200 K. The PIN gradient homojunction show decent performance even at room temperature where $V_{oc}^{300K} = 9.5\;mV$ as showed in Fig. 3b. The IR light using a blackbody radiation source at 600 °C. The incident blackbody radiation power is 134 μW/mm^2^ with the spectra edge at 4.4 μm (~2300 cm^−1^) and 95 μW/mm^2^ with the spectra edge at 3.2 μm (~3100 cm^−1^).

On the other hand, the gradient structure also would increase photocurrent. With zero bias, the photocurrents are 14.7, 8.5, and 10.2 μA for PIN, PI, and IN homojunctions, respectively. The area for all devices is 0.2 mm × 0.2 mm. Since the radiation power density is ~134 μW/mm^2^ for the photoresponse spectra with the cut-off at 4.2 μm. Responsivity $\Re$ would be 2.7, 1.6, and 2.0 A/W for PIN gradient, PI, and IN homojunctions at 80 K. In photovoltaic detectors, the total device photocurrent comes from generated current and diffusion current. Generated current comes from electron-hole pairs risen in the space charge region (SCR), which would be immediately separated by an internal electric field and swept to the electrodes. However, the pairs produced outside the SCR would diffuse, while part of them would be able to reach the SCR and undergo charge separation, giving rise to a diffusion current. The intrinsic CQD solid combined with a matched doped layer would benefit in a much thicker SCR where more photons could be absorbed, while the enhanced internal electric field helps generated carrier pairs to separate. In addition, we compare high-mobility and low-mobility devices (Fig. S3, SI), approving the high mobility would also improve the possibility of diffusing carriers reaching SCR and being collected, where diffusion current would also increase. Figure 3c shows the responsivity as a function of temperature. The responsivity on PIN gradient homojunction drops much slower with temperature increasing, which is still above 1 A/W until 240 K. In contrast, the PI and IN junction show rapid decay above 170 K, which is also relevant to the open circuit voltage variation and observed before. The gradient PIN junction basically benefits HOT mid-IR responsivity.

### Detectivity

The specific detectivity would be a more ideal value to characterize the detector performance, given by

2 $$D^ \ast = \frac{R}{{i_n}}\sqrt A$$

where $i_n$ is the root mean square (rms) current noise in a 1 Hz bandwidth and *A* is the area of the device. Figure 3d, e shows the measured noise spectra at 80 and 300 K, respectively. There would be 1/f noise below 100 Hz, which is possibly a combination of the device itself and the amplifier. The noise level on the flat region is several folds larger compared with the theoretical Johnson noise. At 80 K, for the PIN gradient homojunction, the measured noise $i_n$ is 0.2 pAHz^-1/2^, threefold larger compared to Johnson noise which is $\sqrt {4k_bT/R}$ = 0.06 pAHz^−1/2^, where $k_b$ is the Boltzmann constant 1.38 × 10^−23^ J/K, *T* = 80 K, *R* = 1 M${{\Omega }}$ the resistance linearly fitted by dark current. The specific detectivity in the PIN gradient homojunction device also reaches 2.7 × 10^11^ Jones at 80 K. As shown in Fig. 3f, all high mobility homojunctions show BLIP at cryogenic temperature. However, detectivity on the PI and IN junction would drop quickly at higher temperatures as shown in Fig. 3f. The PIN gradient homojunction shows D* still above 10^11^ Jones until 200 K, where the BLIP temperature increases from 120 to 200 K. The PIN gradient homojunction shows D* still above 10^10^ Jones until 280 K. The response speed is fast, as shown in Fig. 3g. Photoresponse spectra of homojunction device would red-shift while cooling, with an edge at 3.5 μm (~2815 cm^−1^) at 300 K and 4.2 μm (~2400 cm^−1^) at 80 K, showed in Fig. 3h. The spectral response is much sharper compared to absorption, which is possibly from the resonance enhancement. The COMSOL simulation shows that the mid-IR photoresponse would be enhanced with thickness ~1/10 wavelength (Fig. S8, SI). Figure 3i shows dark current as a function of temperature on the PIN gradient device. We compared the dark current with the PI homojunction, approving that the dark current is suppressed in the PIN gradient homojunction (Fig. S4, SI). Obviously, the working temperature in the PIN gradient homojunction device is much improved. The input power from the 600 °C would change from 134 μW/mm^2^ with the spectra edge at 4.2 μm (~2400 cm^−1^) to 95 μW/mm^2^ with the spectra edge at 3.5 μm (~2815 cm^−1^). For the PIN junction, the photocurrent is 2.2 μA, $\Re$ = 0.58 A/W, dark resistance 20 k${{\Omega }}$, theoretical noise 0.9 pAHz^−1/2^, measured noise 1.5 pAHz^−1/2^, and D^*^ = $7.6 \times 10^9$ Jones at 300 K. Table 1 compares the mid-IR photodetection devices with previous reports. We suppose that stronger junctions would further benefit the room temperature performance in homojunction devices. Still, the PIN gradient homojunction has already demonstrated HOT mid-IR photodetection.

**Table 1:** Comparison of typical mid-IR QD photodetectors

Device	Wavelength nm	Bias V	T K	D^*^ Jones	ℜ A/W	ref
(epitaxial) PV/GaAs substrate/n^+^ GaAs /GaAs/ n^+^ InAs QD/ GaAs barrier/ AlGaAs barrier/ GaAs contact	~4300	2	100	10^11^	/	^4^
			170	10^10^	0.12	
(epitaxial) PV/ InAs QD/ AlGaAs barrier	~5000	2	100	2 × 10^9^	0.2	^48^
(epitaxial) InP substrate/InAs QD	~4100	5	120	2.8 × 10^11^	/	^49^
			300	6.7 × 10^7^	/	
(epitaxial)PV/ Si substrate/Ge QD/Si	3000~5000	2	90	8 × 10^10^	0.25	^50^
(colloidal) PC/HgTe QD with EDT	~5000	5	80	6.1 × 10^8^	/	^12^
			200	2.5 × 10^8^	/	
			295	5 × 10^7^	0.1	
(colloidal) PC/ HgTe QD with As_2_S_3_	~3500	3	100	3.5 × 10^10^	/	^14^
			200	2.37 × 10^10^	0.052	
			300	1.54 × 10^9^	0.01	
(colloidal)PV/CaF_2_/NiCr/HgTe QD/Ag	~3700	0	90	4.2 × 10^10^	0.08	^17^
			200	1 × 10^9^	0.01	
			290	1.2 × 10^7^	0.001	
(colloidal) PV/ITO/HgTe QD /Ag_2_Te QD /HgCl_2_/Au	~3700	0	80	1 × 10^11^	0.4	^18^
			200	1 × 10^10^	0.46	
			295	3 × 10^8^	0.08	
(colloidal) PV/ITO/metal disks/ HgTe QD /Ag_2_Te QD / Optical spacer/Au	~4400	0	85	4 × 10^11^	1.6	^20^
			200	1.5 × 10^10^	/	
			300	7.2 × 10^8^	/	
(colloidal) PC /HgTe QD with hybrid ligands	~3770	1.5	80	5.4 × 10^10^	0.2	^13^
			200	2 × 10^9^	/	
			300	6 × 10^7^	/	
(colloidal) PV/ HgTe QD	~4400	0	80	2.7 × 10^11^	2.7	This work
			200	10^11^	2.0	
			280	10^10^	0.6	
			300	7.6 × 10^9^	0.58	

### Quantum efficiency

Naturally, there would be lower quantum efficiency in QD photodetector compared with bulk material photodetector because of the dense packing. Here, the external quantum efficiency

3 $${{{\mathrm{EQE}}}} = \frac{{1.24\Re }}{{\lambda \mu {{{\mathrm{m}}}}}}$$

The EQE in PIN gradient homojunction reaches more than 77% at 80 K, comparable to epitaxial growth HgCdTe detector^28^ in the same wavelength. The spectral dependence of the absorption coefficient has a decisive influence on quantum efficiency^37^. The real part of the optical index for HgTe CQD before and after phase transfer would be *n*_*0*_ = 2.20 ± 0.10 and *n*_*0*_ = 2.95 ± 0.06, measured by ellipsometer^38^. The imaginary part *k* would be fitted by absorption spectra (Fig. S5, SI). We note here the *k* value is not a constant but varies with wavelength. We use the *k* value at ~3.2 μm, which is about the middle of the CQD absorption edge at room temperature. As a result, the *k* value would be 0.1 and 0.2 for low mobility and high mobility CQD solid, respectively. Both *n*_*0*_ and *k* values are increased after CQD phase transfer. We suppose this is due to the much higher packing density. The CQD packing factor could be extracted based on Maxwell-Garnett (MG) model^39^, $\frac{{\varepsilon _{{\mathrm{eff}}} - \varepsilon _{{\mathrm{CQD}}}}}{{\varepsilon _{{\mathrm{eff}}} + 2\varepsilon _{{\mathrm{CQD}}}}} = (1 - f_{{\mathrm{packing}}})\frac{{\varepsilon _m - \varepsilon _{{\mathrm{CQD}}}}}{{\varepsilon _m + \varepsilon _{{\mathrm{CQD}}}}}$, where ε_eff_ is the effective dielectric constant, ε_CQD_ the dielectric constant of CQDs, and ε_m_ the dielectric constant of medium, $f_{{\mathrm{packing}}}$ the packing factor. For high mobility HgTe CQD solid, ε_CQD_ is taken as 15.1^40^. *ε*_*m*_ is taken to be 1, since the CQDs are surrounded by air. Since *n*_*0*_ is much larger than *k*, $\varepsilon _{{\mathrm{eff}}}$ could be estimated as *n*_*0*_^*2*^. $f_{{\mathrm{packing}}}^{{\mathrm{high}}\;{\mathrm{mobility}}}$ is 66 ± 4%, comparable to CQD after imprinting^21^ or with hybrid ligand exchange^31^. This value is much larger than the low-mobility HgTe CQD solid’s packing factor $f_{{\mathrm{packing}}}^{{\mathrm{low}}\;{\mathrm{mobility}}}$= 35 ± 4% calculated with the same theory. (Fig. S6, SI) The packing density could increase by a factor of 1.88 ± 0.2 after ligand exchange, comparable to the factor of 2 increase in *k* value.

Natively, we think the additional improvement in quantum efficiency is due to the improved photoionization from high mobility. The quantum efficiency for photoionization $\eta = \frac{{\tau _R}}{{\tau _R + \tau _{{\mathrm{hop}}}}}$, where $\tau _R$ is the competing exciton recombination rate that includes radiative and nonradiative processes inside of the dot, $\tau _{{\mathrm{hop}}}$ the hopping time, which is directly related to mobility. $\tau _{{\mathrm{hop}}}$ is relevant for optical applications where the exciton needs to be ionized, since if the exciton binding energy is small compared to the thermal energy, its ionization time is simply the hopping time. The relation between the hopping time $\tau _{{\mathrm{hop}}}$ and mobility *μ* could be estimated by Einstein’s relations on diffusion in three dimensions $\mu = \frac{{ed^2}}{{6\tau _{{\mathrm{hop}}}k_bT}}$, where *e* is the elemental electron charge, *d* the CQD diameter, *k*_*b*_ Boltzmann constant, *T* temperature. With CQD diameter of 9 nm, it gives $\tau _{{\mathrm{hop}}}$ ≈ 6 ns for mobility of 10^–3^ cm^2^ V^−1^ s^−1^ while $\tau _{{\mathrm{hop}}}$ ≈ 6 ps for a mobility of 1 cm^2^ V^−1^s^−1^ at room temperature (Fig. S7, SI). In the visible and near-infrared, the radiative recombination would be typically several tens of nanoseconds for CdSe CQDs^41^ and in the microsecond regime for near-infrared PbSe CQDs^42^. Therefore, mobility of 10^−3^–10^–2^ cm^2^ V^−1^ s^−1^ in the visible and near-infrared CQD should already give ionization efficiencies larger than 99%. However, for applications in the SWIR and MWIR, energy transfer to the ligands or matrix phonons may be more rapid than 1 ns^12^, where mobility closer to 1 cm^2^ V^−1^ s^−1^ will be required to achieve high ionization efficiencies near 100%.

What’s more, with the measured absorbance and film thickness, we find the absorption coefficient of dense-packing mid-IR CQD reaches several thousand cm^−1^ for band edge in the mid-IR spectral range. The value is comparable to typical mid-IR bulk^1^, which is around 10^4^ cm^−1^. The absorption coefficient indicates the CQD solid thickness of several hundred microns would be enough to maximize quantum efficiency. (Fig. S8, SI).

The improved EQE also comes from the high carrier collection efficiency as a result of high mobility and homojunction design. We compared high mobility homojunction with heterojunction, where carrier mobility mismatching at the interface causes more than 50% photocurrent loss (Fig. S9, SI).

### Applications

**Fig. 4:**
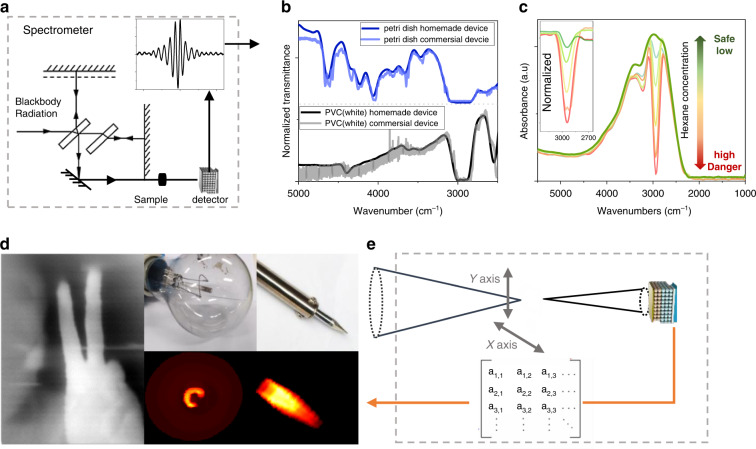
Homojunction device application. **a** Schematic diagram of the spectrometer with a homojunction device. **b** Transmission measured by homojunction device and commercial FTIR. **c** absorption with different hexane concentrations in the air. **d** Thermal imaging. **e** Schematic diagram of one-pixel imaging set-up

With the Michelson interferometer, the mid-IR CQD detector could be served as a spectrometer, as shown in Fig. 4a. The infrared light that comes out from the Michelson interferometer is reflected by a gold mirror, then absorbed by chemical samples before being collected by the photodetector, like normal Fourier Transform infrared spectroscopy (FTIR). The interference is then collected by a computer and processed with a fast Fourier Transform (FFT). Figure 4b shows the transmission spectra of a piece of a petri dish and white polyvinyl chloride measured by FTIR and our homemade spectrometer, where all signatures could be figured out.

The detector could also be applied as a gas sensor^43,44^ Here, we take hexane as an example. The homemade spectrometer is put in the fume hood. With pure air, the absorption spectrum is the green line, while absorption of the atmosphere with different hexane concentrations is measured as shown in Fig. 4c. One drop of hexane (0.05 ml~0.4 mmol) evaporated in the fume hood is the pale green line while 10 drops of hexane (0.5 ml) is a red line. Since the air is ~24 L/mol with the hood ~2000 L, one could estimate hexane concentration varies from 4.8 ppm to 48 ppm. The C-H bond vibration cause strong absorption ~3000 cm^−1^. Taking this as the reference, whenever the relative photoresponse value~3000 cm^-1^ is below 0.1, this gas sensor would regard the atmosphere in the hood as harmful and alarm, which is an easy, useful, and interesting application. Thermal images are also successfully taken by a one-pixel HgTe CQD infrared camera (Methods), as shown in Fig. 4d, which could figure out the filament shape in tungsten lamp, the temperature difference inside the soldering iron tip as well as the human fingers.

Overall, we introduce a modified ligand exchange method on min-IR CQD, which separates mobility improvement, doping control and Fermi level fixing in different steps, providing precise control of transport properties. The controllable transport properties combined with solution processability could benefit the easy fabrication of the gradient homojunction photovoltaic detector, which shows more than 50% larger open circuit voltage and responsivity compared with the reference sample. The device achieves BLIP detection above 10^11^ Jones until 200 K and above 10^10^ Jones until 280 K, approving a HOT device on mid-IR photodetection. What’s more, the dense CQD packing would also enhance the infrared absorption of the CQD layer, achieving an EQE of 77%, a several-fold increase compared to the previous pure solid ligand exchange CQD solid with a similar thickness. Applications like spectrometers, chemical sensors as well as infrared cameras, are approved, indicating the possible low-cost but sensitive alternatives to epitaxial commercial photodetectors for applications in daily life.

## Materials and methods

### Synthesis of colloidal nanomaterials

The synthesis of HgTe CQDs following reported reference. HgCl_2_ (0.15 mmol) is dissolved in 4 g of oleylamine(OAM) in a 20 mL glass vial at 100 °C for 60 min with stirring in the glovebox. Bis(trimethylsilyl)telluride (TMSTe) (98%) was purchased from Acros and stored inside the freezer in a nitrogen glovebox. About 100 μL TMSTe is diluted in 1 mL OAM, and rapidly injected into HgCl_2_ precursor. The clear solution immediately turns black. The reaction is quenched by injecting 4 mL tetrachloroethylene (TCE).

### Modified mixed-phase ligand exchange method

The mixed-phase ligand exchange process involves three steps, including liquid phase ligand exchange, doping modification by additional salts, and solid-phase ligand exchange. In the liquid phase ligand exchange, 10 ml HgTe CQD in hexane would be mixed with 200 μL β- mercaptoethanol (β-ME) in *N*,*N*-Dimethylformamide (DMF), 5 mg dimethyldioctadecylammonium bromide (DDAB) could be added to accelerate phase transfer process. For typical p-type HgTe CQD, 5 mg (NH_4_)_2_S would be added in CQD/DMF. For the typical n-type, 20 mg HgCl_2_ salt would be added. For the very intrinsic CQD, 10 mg HgCl_2_ salt would be added.

CQD were precipitated by adding toluene as the anti-solvent, followed by centrifugation. XPS of the CQD surface with different doping is included (Fig. S10, SI). After discarding the supernatant, 40 µL DMF was used to dissolve the CQD solids, yielding a colloidally-stable solution. The CQD films are prepared by spin-coating and followed by solid-phase ligand exchange with ethanedithiol (EDT)/HCl/IPA (1:1:50 by volume) solution for 10 s, rinsed with IPA and dried with N_2_. EDT is like β-ME but with two thiols and a stronger attaching ligand for HgTe CQDs^45^^,^ while HCl would stabilize the Fermi level in the CQD solid^33^. What’s more, the similar ligand length between β-ME and EDT won’t introduce cracks compared to pure solid ligand exchange^46,47^. Solid-phase ligand exchange is necessary to remove the extra hybrid ligands, which also fixes the Fermi level of the CQD films. Electrochemistry is used to figure out the energy diagram (Fig. 11, SI). We note the CQD solution after phase transfer is called CQD ink.

### Cleaning procedure for the QD

Put the synthesized HgTe quantum dots into the centrifuge tube and ~30 ml IPA (Isopropanol) was added for cleaning, stirred and put into a centrifuge. After 7500 r/min for 6 min, centrifugation and precipitation. the upper clear liquid was discarded, and the precipitated quantum dots were dried with nitrogen, and then the quantum dots were dissolved with 10 ml of n-hexane.

### Device fabrication

For the PI homojunction device, the detector is fabricated on an Al_2_O_3_ substrate. About 50 nm ITO on deposited on the substrate by magnetron sputtering and annealed at 300 °C for 15 min before drop-casting the colloidal ink. First, the high mobility intrinsic HgTe CQD ink was drop-cast into a film with a thickness of about 340–350 nm. Then, 120 nm p-type high mobility HgTe CQDs are fabricated. Finally, 50 nm Au was deposited as the top contact. For PIN gradient homojunction, ~60 nm thickness n-type (doping at ~0.8 e/dot), ~40 nm thickness n-type (doping ~0.1 e/dot), ~300 nm thickness intrinsic, ~40 nm p-type (doping ~0.1 h/dot), and ~60 nm p-type (doping ~0.8 h/dot) CQD solids are stacked. For IN homojunction, ~350 nm thickness intrinsic, ~120 nm n-type (doping ~0.8 h/dot) CQD solids are stacked.

### Field effect transistor

Silicon wafers with dry thermal oxide (n^++^ Si/300 nm SiO_2_) were used as the substrates. The FET mobility ($\mu ^{{\mathrm{FET}}}$), extracted in the linear regime, was calculated by fitting the transfer curve to the following equation: $\mu ^{{\mathrm{FET}}} = \frac{L}{{WC_iV_D}}\frac{{dI_D}}{{dV_g}}$, where *L*, *W*, *C*_i_, *V*_D_, *I*_D_, and *V*_g_ are the channel length, channel width, capacitance per unit area, drain voltage, drain current, and gate voltage, respectively. We also verified that in all measurements FET channel current exceeded the gate leakage current by several orders of magnitude.

Electrode design: Four pairs of interdigitated evaporated gold electrodes with a finger width of 10 microns, a gap of 10 microns (channel length in FET), and finger length of 300 microns (channel width in FET).

Carrier density is estimated by $\Delta V_gC_i/N$, where $\Delta V_g$ is the absolute gate potential where the film conductivity reaches a minimum in FET measurement result, N the CQDs areal density ($\propto d^{ - 2}$, *d* the CQD diameter). Take p-type HgTe CQD solid as an example, $\Delta V_g$ = 13 V, CQD average diameter ~9 nm, $C_i$ = 1.15 × 10^-4^ F/m^2^. Therefore, the FET indicates 0.8 $\pm 0.12$ holes per dot.

### Photodetector characterization

All photovoltaic devices are placed in a closed-loop cryostat for temperature-dependent characterization. The effective homojunction area is 0.2 mm × 0.2 mm. The IR light uses a blackbody radiation source at 600 °C. The incident blackbody radiation power is 134 μ W/mm^2^ with the spectra edge at 4.2 μm and 95 μ W/mm^2^ with the spectra edge at 3.2 μm. The IV-curves are recorded by source meter Keithley 2602B. Noise spectra is recorded by Noise analyzer SR770 (Stanford Research Systems). For response speed, the incident light is modulated by a chopper at 500 Hz, the photocurrent is recorded by an oscilloscope.

#### Spectral response

As shown in Fig. 4a, the detector was placed inside a vacuum cryostat. The calibrated blackbody combined with Michelson interferometry is used as the light source. The electric output from the detector is amplified by an amplifier, with a gain of 107 V/A (bandwidth of 500 kHz). In the Michelson interferometry, the velocity of a mobile mirror is 0.4747 cm/s (no obvious difference if the velocity change from 0.4747 to 0.96 cm/s). The interference peak signal is recorded in the time domain, as shown in Fig. 4a. The photodetector position is adjusted where the interference peak is relatively high. About 25–30 sets of interference peak data are collected through the oscilloscope. Each data were processed using a Fast Fourier transform. The wavenumber can be obtained from the ratio of the frequency to the active mirror velocity.

### Spectrometer and gas sensing

The photovoltaic device is placed in a closed-loop cryostat for temperature-dependent characterization. The blackbody radiation after the Michelson interferometer could be detected by the CQD photodetector. The electric signal from the photodetector would be recorded by an oscilloscope. Then, the Fourier transform would be done to obtain the photoresponse spectra. For the spectrometer, a piece of the petri dish and white polyvinyl chloride would be put in front of the cryostat window. For the gas sensor, since the light travel through the atmosphere in the fume hood, the gas concentration difference could be figured out on the photoresponse spectra.

### Ellipsometry measurement

For the ellipsometry measurement, we used the Gaertner Waferskan Ellipsometer Model L116S. Phase-transferred HgTe CQD films were prepared on the Si chips (area: 1 inch × 1 inch, thickness: 1 mm). During the measurement, the HeNe 6328 Angstrom Laser provided less than 1 mW output on a sample with a 1 mm beam diameter at a 70° incidence angle. The detector and analyzer then received the reflection and characterized the change of polarization parameters like amplitude ratio and the phase difference. The optical index was calculated from the polarization parameters.

### Single-pixel scanning imaging

The imaging system consisted of five parts: scanning lens, cryostat, HgTe CQD detector, amplifier, and software. Controlled by the software, a BaF_2_ lens (f = 30 mm, 1451 A LBTEK) is scanned over a 15 × 15 mm^2^ area. Two linear motorized stages are used to scan the lens. As the projected image of the object moves over the detector, the photocurrent is amplified and sampled by the software at a 3 kHz sampling rate. The recorded data array is then used to construct images. The numbers of points in the vertical and horizontal directions are 75 and 500, respectively.

## Supplementary information

Supplemental material
