# Supplementary material for: High-operating-temperature mid-infrared photodetectors via quantum dot gradient homojunction
Source: Light Sci Appl. 2023 Jan 1;12:2. doi: 10.1038/s41377-022-01014-0 (PMC9805449; doi:10.1038/s41377-022-01014-0)
Supplement: Supplementary file 1 — Supplemental material [file 41377_2022_1014_MOESM1_ESM.docx]

**Supplementary Information for**

**High-Operating-Temperature Mid-Infrared Photodetectors *via* Quantum Dot Gradient Homojunction**

*Xiaomeng Xue^1#^, Menglu Chen^1,2,3#*^, Yuning Luo^1^, Tianling Qin^1^, Xin Tang^1,2,3*^, Qun Hao^1,2,3*^*

1. School of Optics and Photonics, Beijing Institute of Technology, No. 5 Zhongguancun South Street, Beijing, China.
2. Beijing Key Laboratory for Precision Optoelectronic Measurement Instrument and Technology, Beijing, China.
3. Yangtze Delta Region Academy of Beijing Institute of Technology, China.

^#^These authors contributed equally to this work

^*^Corresponding authors: [menglu@bit.edu.cn](mailto:menglu@bit.edu.cn); xintang@bit.edu.cn; qhao@bit.edu.cn

1. **Solid ligand exchange benefit doping stability**
2. **State resolved transport at 80 K**
3. **Low mobility PI homojunction**
4. **Dark current comparison**
5. **Material absorption and index fitting with COMSOL simulation**
6. **Packing density estimation**
7. **Discussion on hopping time and mobility**
8. **Calculation of IQE**
9. **High mobility PI heterojunction**
10. **XPS elements analysis**
11. **Energy diagram**

**1. Solid ligand exchange benefit doping stability**

**Figure S1** shows the benefits from 1,2-Ethanedithiol/HCl (EDT/HCl) ligands. **Figure S1a** shows absorption spectra with liquid phase ligand exchange with 2-Mercaptoethanol (β-ME) and after solid ligand exchange with EDT/HCl. One could see the residue ligands are much removed from the O-H and C-H oscillation. **Figure S1b** shows FET transfer curves of CQD solid immediately after ligand exchange and after 1 week exposed to air with β-ME only and with EDT/HCl, respectively. One could see the solid would be more intrinsic and more stable in the ambient environment after EDT/HCl treatment.


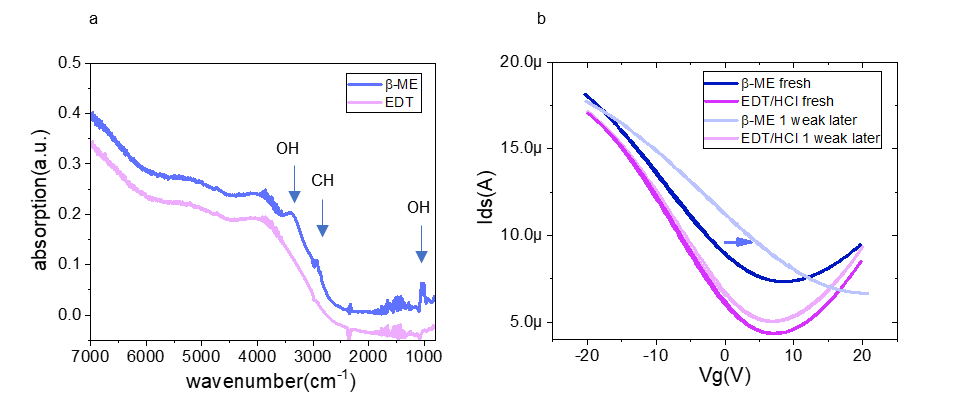


**Figure S1. Benefits from EDT/HCl ligands.** (a) absorption spectra with liquid phase ligand exchange with β-ME(Blue) and after solid ligand exchange with EDT/HCl(pink). (b) FET transfer curves of CQD solid immediately after ligand exchange and after 1week exposed to air.

**2. State resolved transport at 80 K**

State resolved transport in mid-infrared HgTe CQD could be observed with cooling. **Figure S2** shows the transfer curves of p type, intrinsic and n type HgTe CQD at 80 K on linear and log scale. The pumps would be electrons filling the lowest state 1S_e_ in the conduction band.

**
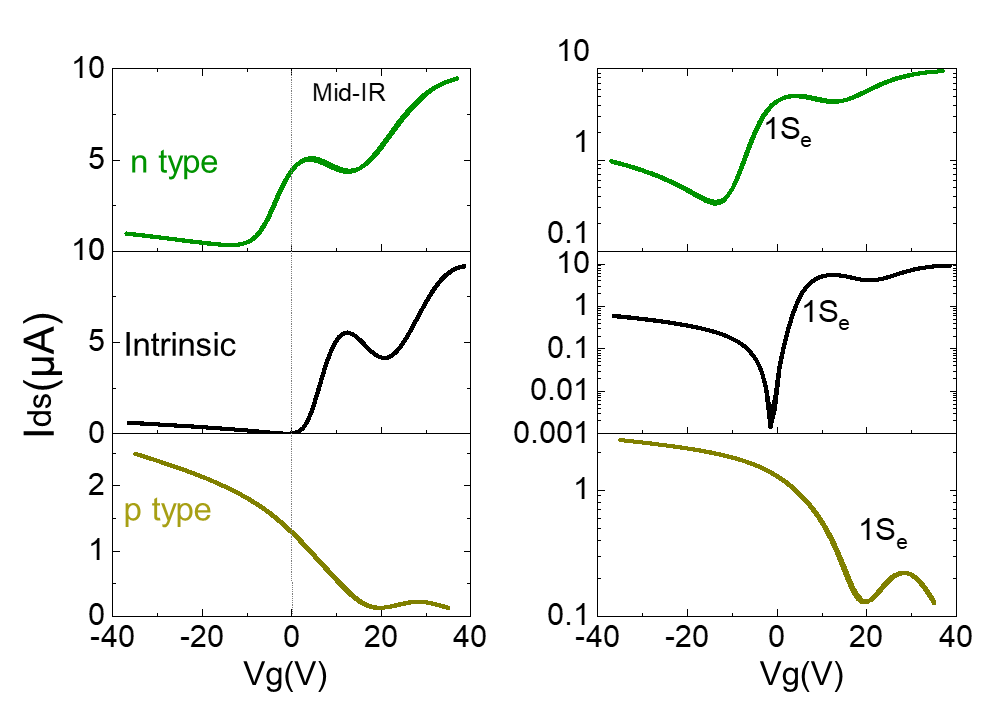
**

**Figure S2. State resolved transport at 80 K**

**3. Low mobility PI homojunction**

For low mobility device, ~350 nm HgTe CQDs caped with OAM was drop-cast on the ITO/ Al_2_O_3_ substrate and treated with ligand-exchange with EDT/HCl/IPA (1:1:50 by volume) solution for 10 s, rinsed with IPA and dried with N_2_. Then, ~120 nm HgTe CQDs was drop-cast treated with ligand-exchange of 1 mM (NH_4_)_2_S/MeOH. Finally, 50 nm Au was deposited as the top contact.


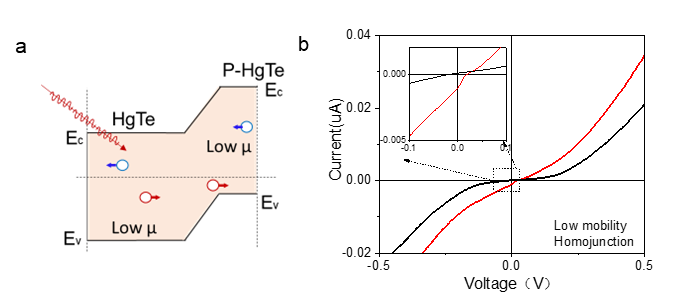


**Figure S3. Low mobility PI homojunction**

The energy diagrams are shown in **Figure S3a**. The IV curve characterization at 80 K of different junctions are shown in **Figure S3b**, where open circuit voltage is 20 mV and photocurrents without bias is 0.0014 μA, approving the junction benefits from high mobility.

**4. Dark current comparison**

**Figure S4** shows dark current comparison of PI and PIN gradient homojunctions as a function of temperature from 80 K to 300 K.


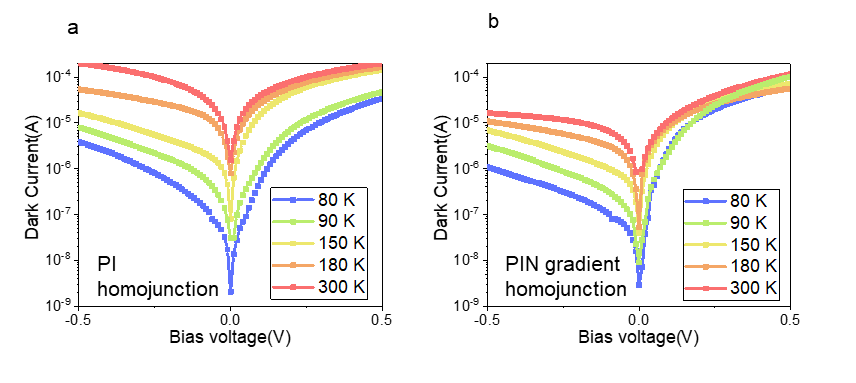


**Figure S4. Dark current comparison of PI and PIN gradient homojunction.**

**5. Material absorption and index fitting with COMSOL simulation**

Measured absorption by FTIR of 400nm CQD on ZnSe window is showed in **Figure S5**. COMSOL simulation of absorption is also showed with parameters that real part of optical index *n_0_* is 2.95±0.6, solid thickness 400nm, and varying *k*=0.15(pale blue),0.20 (blue), and 0.25(dark blue). So, *k*=0.20 is chosen for this close packing solid.

For quantum dot, the absorption$\alpha=\frac{4\pi k}{\lambda}$, which could be expressed as $\alpha(\hbar\varpi)=\alpha_{0}\frac{n_{e}}{\delta}\frac{\sigma_{QD}}{\sigma_{ens}}exp[-\frac{{(\hbar\varpi-E_{g})}^{2}}{\sigma_{ens}^{2}}]$, $\alpha_{0}$ is the maximum absorption coefficient, $n_{e}$ the areal density of electrons in the quantum dot ground state, $\delta$ the quantum dot density, $\sigma_{QD}$ the energy distribution of single QD and $\sigma_{ens}$ the energy distribution of the QD ensemble, $E_{g}$ the energy gap. $\frac{hc}{\lambda}=\hbar\varpi$. We mimic the real absorption with several gaussians.


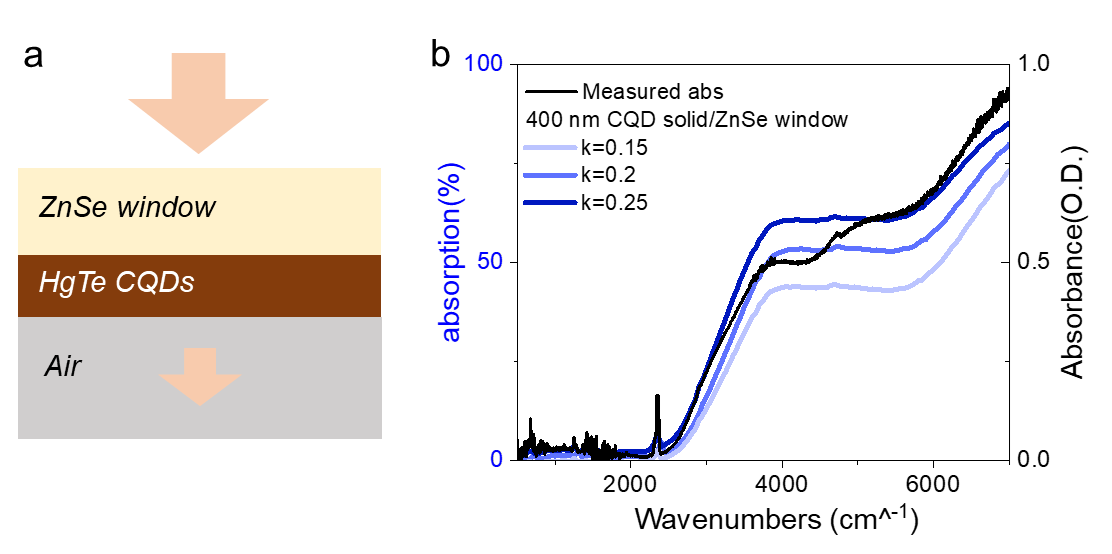


**Figure S5. Optical index k fitting.** Measured absorption by FTIR of 400nm CQD on ZnSe window (Black line). COMSOL simulation of absorption with n fixed at 2.95, thickness 400nm, and varying k=0.15(pale blue),0.20 (blue), and 0.25(dark blue).

**6. Packing density estimation**

Based on Maxwell-Garnett model, $\frac{\varepsilon_{eff}-\varepsilon_{CQD}}{\varepsilon_{eff}+2\varepsilon_{CQD}}=(1-f_{packing}) \frac{\varepsilon_{m}-\varepsilon_{CQD}}{\varepsilon_{m}+\varepsilon_{CQD}}$, where ε_eff_ is the effective dielectric constant, ε_CQD_ the dielectric constant of CQDs, and ε_m_ the dielectric constant of medium, $f_{packing}$ the packing factor. For high mobility HgTe CQD solid, ε_CQD_ is taken as 15.1. ε_m_ is taken to be 1, since the CQDs are surrounded by air. Since *n_0_* is much larger than *k*, $\varepsilon_{eff}$ could be estimated as *n_0_*^2^. The low mobility solids have measured optical index *n_0_*=2.2±0.1 for MWIR while the high mobility solids have measured optical index *n_0_*=2.95±0.06 for MWIR for by ellipsometer. The packing factor $f_{packing}$ estimated by Maxwell-Garnett model is showed in **Figure S6**. The packing factor $f_{packing}$ would be 35±4 % for low mobility and 66±4% for high mobility solids, respectively. As a result, the packing density increased a factor 1.88 ± 0.2.

For the ellipsometry measurement, we used the Gaertner Waferskan Ellipsometer Model L116S. Phase transferred HgTe CQD films were prepared on the Si chips (area: 1 inch *1 inch, thickness: 1mm). During the measurement, the HeNe 6328 Angstrom Laser provided less than 1 mW output on sample with 1mm beam diameter at 70° incidence angle. The detector and analyzer then received the reflection and characterized the change of polarization parameters like amplitude ratio and the phase difference. The optical index was calculated from the polarization parameters.


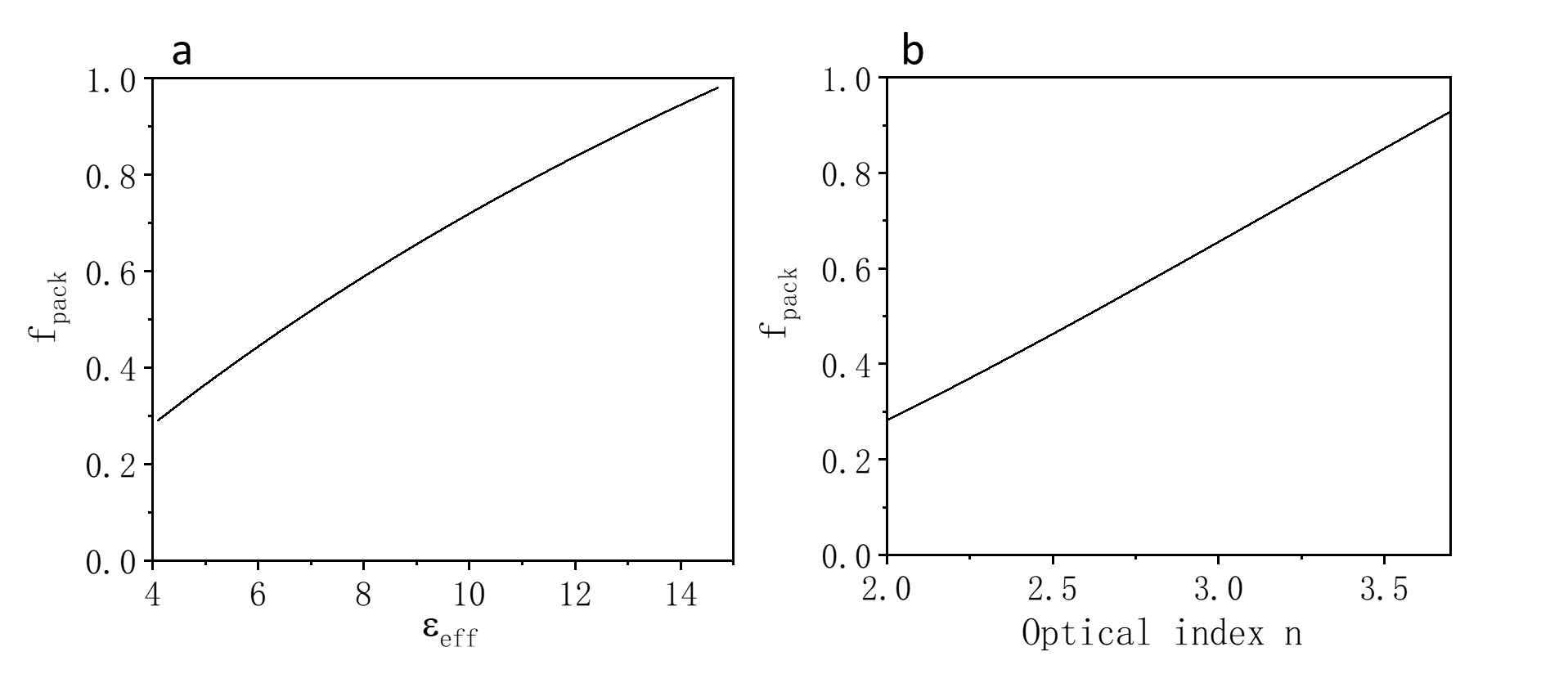


**Figure S6. (a, b) Maxwell-Garnett model fit of the packing factor** $\boldsymbol{f}_{\boldsymbol{packing}}$**.**

1. **Discussion on hopping time and mobility**

The relation between the hopping time $\tau_{hop}$ and mobility $\mu$ could be estimated by Einstein’s relations on diffusion in three dimensions $\mu=\frac{ed^{2}}{6\tau_{hop}k_{b}T}$, where e is electron charge, d the CQD diameter, k_b_ Boltzmann constant, T temperature. With CQD diameter ~9 nm. it gives $\tau_{hop}$ ≈ 6 ns for a mobility of 10^–3^ cm^2^V^-1^s^-1^ while $\tau_{hop}$ ≈6 ps for a mobility of 1 cm^2^V^-1^s^-1^ at room temperature as showed in **Figure S7**.

**Figure S7. Hopping time and mobility**

1. **Quantum efficiency**

The COMSOL simulation is also used to discuss how would the n and k factor value effect on device absorption. **Figure.S8a** illustrated the structure of model which is the general device design. **Figure S8b** shows how spectral optical absorption abs(ω) varies with different device thickness with fix *k*=0.20, *n*=2.95. We assume 90% reflection from Au contact, where the black line is the reference without Au reflect layer. The total absorption could reach more than 97%, which is the upper limit for external quantum efficiency.

The internal quantum efficiency $IQE=\frac{I_{ph}/e}{\varphi_{abs}}$, where $I_{ph}$ is the photocurrent, e the elementary charge, $\varphi_{abs}$ the absorbed photon flux. Here $\varphi_{abs}$ is simulated by COMSOL Multiphysics.


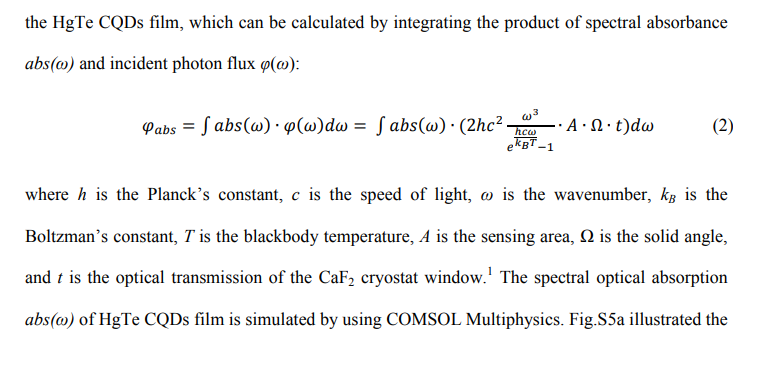
Where spectral optical absorption abs(ω) of HgTe CQDs film, where h is the Planck’s constant, c is the speed of light, ω is the wavenumber, k_B_ is the Boltzmann’s constant, T is the blackbody temperature, A is the sensing area, Ω is the solid angle, and t is the optical transmission of the CaF_2_ cryostat window. Then our IQE achieve more than 90%.


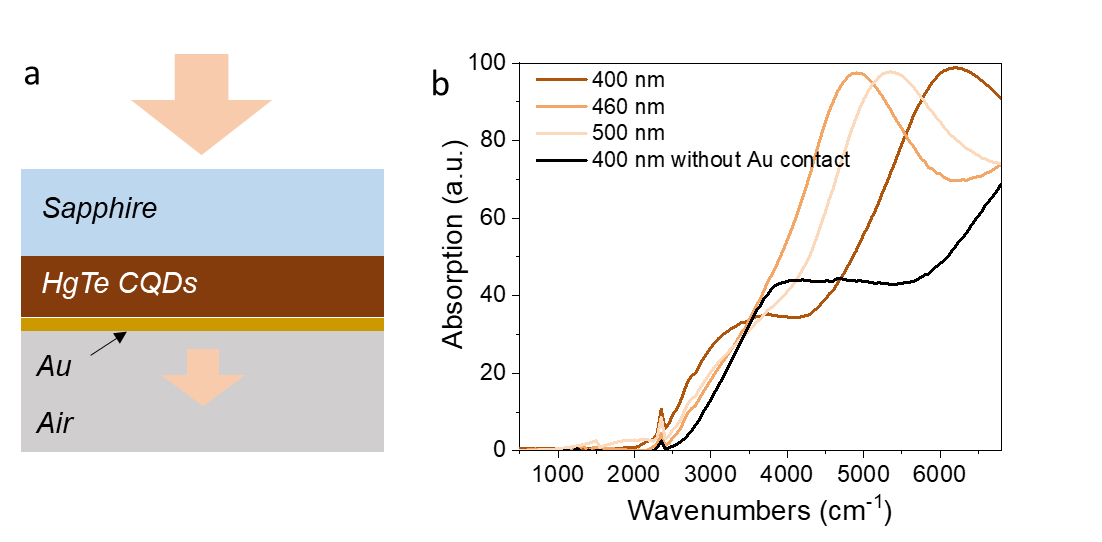


**Figure S8. COMSOL simulation.** a. structure of model. b. Total device absorption varies with CQD thickness.

1. **High mobility PI heterojunction**

For heterojunction device, ~450 nm high mobility intrinsic HgTe CQD ink was drop-cast on the ITO/ Al_2_O_3_ substrate. Then, a layer of Ag_2_Te nanocrystals was spin-coated on the HgTe CQDs as the p-dopant at 3000 rpm for 60 sec, followed with HgCl_2_ (10 mM) treatment for 10 sec. A second layer of Ag_2_Te nanocrystals was deposited on the first Ag_2_Te nanocrystals layer in the same way but without HgCl_2_ treatment. Finally, 50 nm Au was deposited as the top contact.

For Ag_2_Te nanocrystals, silver(I) nitrate (AgNO_3_, 34 mg, 0.2 mmol) was dissolved in 5mL OAm with 0.5mL oleic acid for 30 minutes with stirring at 70 °C in a nitrogen glovebox. Once dissolved, 0.5mL trioctylphosphine was injected and the clear solution was heated rapidly to 160 °C. The solution began to turn yellow at temperatures above 140 °C and was kept at 160 °C for 35-45 minutes until it was orange colored. A separate solution of 0.1 mL TOP-telluride (TOPTe, 1 M) was injected to the reaction solution. The solution immediately turned from orange to black, and the reaction time was 10 minutes. Then, the reaction mixture was removed, cooled, and stored in a freezer (-8 °C) until required. Prior to being drop-casted, the HgTe CQDs and Ag_2_Te nanocrystals solution are cleaned. The CQD solutions are precipitated with an equal volume of IPA and centrifuged at 4500 rpm for 2 min before it is resuspended in 4 mL of hexane.

The energy diagrams are shown in **Figure S9a**. The IV curve characterization at 80K of different junctions are shown in **Figure S9b**, where open circuit voltage is 30mV and photocurrents without bias is 4.1μA. The homojunction device shows at least 2-fold larger photocurrent compared to heterojunction device. We assume the mismatching in the carrier mobility at the interface might be the reason. In the heterojunction device, carrier mobility in Ag_2_Te CQD layer is 10^-3^~10^-4^ cm^2^V^-1^s^-1^ (**Figure S9c**), much slower than the high mobility HgTe CQD which is above 1 cm^2^V^-1^s^-1^. This low mobility layer may trap the photon-excited carriers.


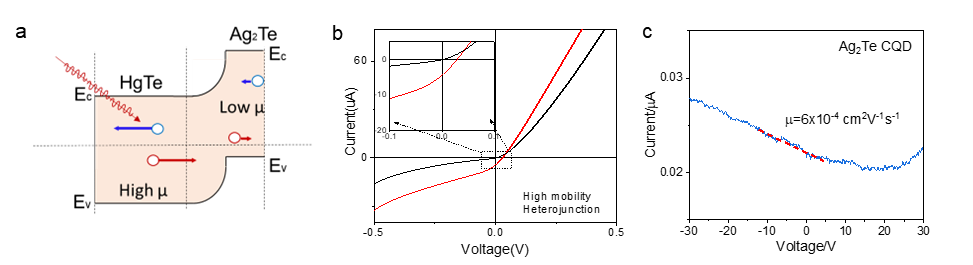


**Figure S9. High mobility PI heterojunction.** (a) energy diagram. (b) IV curve characterization. (c) Ag_2_Te FET transfer curve.

1. **XPS elements analysis**

**Figure S10a** shows the XPS analysis of p type HgTe CQD and intrinsic HgTe CQD solid, where the insert graph shows the 2p state in S element. **Figure S10b-10c s**how the relative Hg to Te ratio increased in the intrinsic HgTe CQD film compared to p type HgTe CQD. This verifies our discussion of the role of Hg^2+^ on CQD doping.

**
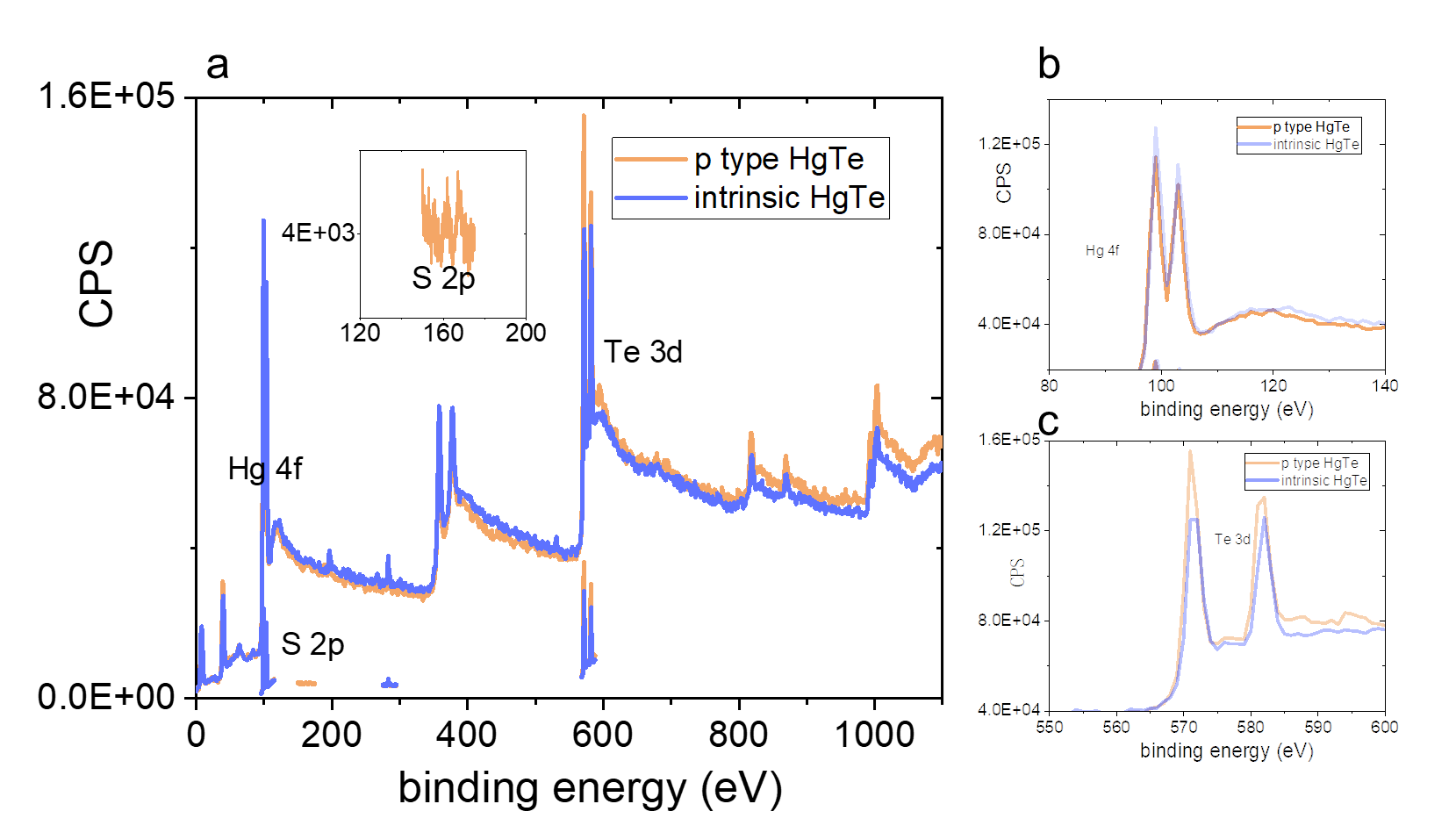
**

**Figure S10. XPS of P type HgTe and intrinsic HgTe.**

1. **Energy scale**

With additional HgCl_2_ or (NH_4_)_2_S salts, the HgTe CQD solids shows n type or p type doping respectively, as showed in **Figure S11a-11b**. Reversible electrochemistry, provides the measurement of the Fermi level E_F_ by rest potential (red arrow) and the absolute measurements of the filled and empty state energies with the application of liquid gate voltages (grey and black lines). The Fermi level is within 50 mV compared to standard calomel electrode (SCE, -4.68±0.02 eV /vacuum) among all HgTe CQD solids, while the energy state would shift ~0.15 eV between n type and p type HgTe CQD solids. n type HgTe ~0.85±0.06 electron per dot(e/dot), p type HgTe ~1.0±0.06 hole per dot(h/dot). The energy diagram is showed in **Figure S11c-11e**, respectively.


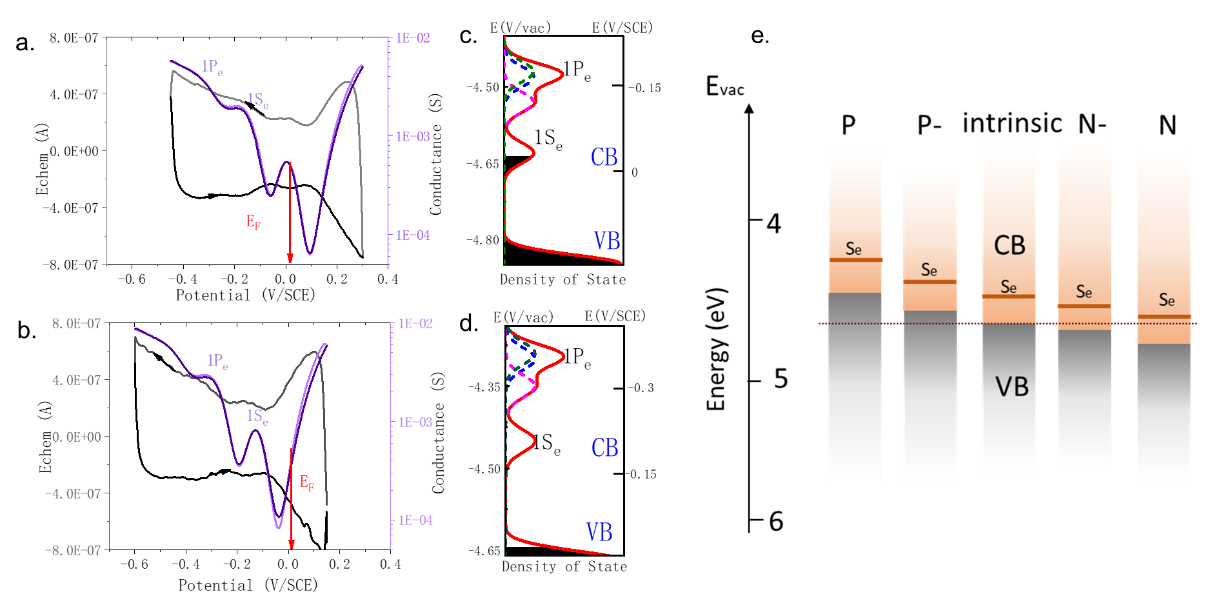


**Figure S11. Energy band.** (a-b) Echem of HgTe CQD solid with n-type doping or p-type doping with doping ~0.8|e|/dot/ at 203K, respectively. The black curve is cyclic voltammetry result, purple curves conductance at different potential with arrow indicating forward and backward scan, red arrow indicating rest potential (Fermi Level). (c-d) Energy diagrams of HgTe CQD solid corresponding to Echem, respectively. Black color indicates that states are filled. (e) Energy diagram on strong p type, weak p type, intrinsic, weak n type, and strong n type HgTe CQD, respectively.
